# Comparison of botulinum toxin and propranolol for essential and dystonic vocal tremors

**DOI:** 10.6061/clinics/2018/e87

**Published:** 2018-06-23

**Authors:** Grazzia Guglielmino, Bruno Teixeira de Moraes, Luiz Celso Villanova, Marina Padovani, Noemi Grigoletto De Biase

**Affiliations:** I Departamento de Otorrinolaringologia e Cirurgia de Cabeca e Pescoco, Universidade Federal de Sao Paulo, Sao Paulo, SP, BR; II Departamento de Cirurgia, Universidade Federal de Pernambuco, Recife, PE, BR; III Departamento de Neurologia, Universidade Federal de Sao Paulo, Sao Paulo, SP, BR; IV Fonoaudiologia, Universidade Federal de Sao Paulo, Sao Paulo, SP, BR; V Departamento de Otorrinolaringologia e Cirurgia de Cabeca e Pescoco, Universidade Federal de Sao Paulo, Sao Paulo, SP, BR; VI Pontifícia Universidade Catolica de Sao Paulo, Sao Paulo, SP, BR

**Keywords:** Botulinum Toxin, Propranolol, Vocal Tremor, Treatment Outcome, Dystonic Tremor

## Abstract

**OBJECTIVES:**

Vocal tremors, which cause social difficulties for patients, may be classified as resting or action tremors. Of the vocal action tremors, essential and dystonic tremors are the most common. Botulinum toxin and oral medications have been used to treat vocal tremors, but no comparative clinical trials have been performed. The aim of this study was to compare the effects of botulinum toxin injection and the oral administration of propranolol in the treatment of essential and dystonic vocal tremors.

**METHODS:**

This clinical trial recruited 15 patients, divided into essential and dystonic vocal tremor groups. Patients in both groups received successive treatment with botulinum toxin and propranolol. The treatments were administered at different times; the order of treatment was randomly selected. Patients were assessed with flexible nasofibrolaryngoscopy and with perceptual and acoustic voice evaluations. A statistical significance level of 0.05 (5%) was used.

**RESULTS:**

Botulinum toxin produced statistically significant improvements in perceptual measures of vocal instability in patients with dystonic vocal tremors compared with baseline values and treatment with propranolol. The acoustic measure of variability in the fundamental frequency was significantly lower in patients with dystonic vocal tremors after treatment with botulinum toxin.

**CONCLUSION:**

Essential and dystonic vocal tremors responded differently to treatment. Dystonic vocal tremors responded significantly to treatment with botulinum toxin but not oral propranolol. Essential vocal tremors did not respond significantly to either treatment, perhaps due to the small number of patients, which is a limitation of this research.

## INTRODUCTION

Vocal tremor is a phonatory, rhythmic voice change characterized by involuntary oscillating movements 1 that can alter vocal production and affect communication, social interaction, and quality of life to a greater or lesser degree depending on the individual.

Essential tremor is the most common type of vocal tremor, followed by Parkinson’s disease and dystonic tremors 2. Essential and dystonic tremors are both action induced but are clinically different 3. Essential vocal tremor is observed in all postures produced by the larynx, both phonatory and non-phonatory 4. Dystonic vocal tremor is phonation dependent and does not occur in non-phonated emissions. These postures and specific tasks should be evaluated by nasofibrolaryngoscopy to classify and differentiate the types of vocal tremors 5,6.

Injection of botulinum toxin into the thyroarytenoid (TA) muscle is the primary treatment choice for dystonia 7. However, a comparable level of treatment success is not observed with essential tremors 3, perhaps because several muscles are involved in this disorder, as has been observed in electromyographic studies 8. The orally administered beta-blocker propranolol is generally poorly effective in treating essential vocal tremors, although it usually decreases or eliminates tremors in the extremities 9-11. Some studies have reported an improvement in essential tremors using botulinum toxin, coinciding with a reduction in laryngeal tremors and supraglottic hyperfunction, as identified with perceptual, acoustic, and aerodynamic analyses 9-12. However, Warrick et al. 13 published a prospective study in which most patients showed no improvements in objective acoustic measures when given botulinum toxin.

No published studies have compared the responses of the two most frequently occurring types of action-induced vocal tremors to treatment with botulinum toxin injection and oral medications. Information regarding the responses to the two therapeutic approaches, along with objectively established clinical diagnoses, will aid in the treatment of patients with vocal tremors. The objective of this study was to verify and compare the responses of patients with essential and dystonic vocal tremors to treatment with botulinum toxin injected into the TA muscle and oral administration of propranolol.

## METHODS

The study was approved by the ethics and research committee (CEP no. 87040/12) and was registered at the Institute of Scientific and Technological Information in Health, identified by the Brazilian Registry of Clinical Trials with the code 389mzn. (http://www.ensaiosclinicos.gov.br/rg/rbr-389mzn). Individuals treated for vocal tremor in the neurolaryngology clinic of a university hospital between January 2012 and August 2013 were included in this randomized clinical trial. All patients read, understood, and signed an informed consent form.

Patients included were over 18 years of age and had been diagnosed with either dystonic or essential vocal tremor. Exclusion criteria were contraindications for the use of beta-adrenergic blockers, laryngeal paralysis or morphological lesions in the phonation apparatus, and signs of Parkinson’s or cerebellar disease.

Twenty-three individuals with vocal tremors were selected for the study. Of these 23 patients, 6 were excluded due to cardiopulmonary contraindications, 1 due to death, and 1 due to adverse effects of propranolol. Of the remaining 15 patients, 10 had dystonic tremor and 5 had essential tremor (Figure 1) 14. Of the 5 patients with essential tremor, 4 presented tremors of the hands, and 4 had a family history of tremors. No patient reported improvements in tremors with alcohol intake, although 3 of them did not use alcohol. The 10 patients with dystonic tremors presented tremors in the palate and larynx only.

All patients underwent nasofibrolaryngoscopy, which was performed without topical nasal anesthesia. Each patient was asked to perform the following tasks: the prolonged emission of phonemes (/ε/ usual tone, /s/, and /z/), quiet breathing, and a continuous whistle. The tasks were performed for at least 5 seconds. The examinations were performed under continuous halogen lighting with a flexible endoscope (FNL-15RP3, Pentax, Tokyo, Japan) connected to a video camera (IK-CU44A, Toshiba, Tokyo, Japan) and were digitally recorded.

The nasofibrolaryngoscopy video images were edited to identify each task performed and to exclude sounds. After editing, the videos were coded and recorded on DVD. The examinations were distributed randomly for recording. Three otolaryngologists with experience in neurolaryngology, who were blinded to the patients’ identities, performed a perceptual-visual analysis of the videos. Diagnoses were made by observing tremors that occurred during vowel production and during the tasks that separated the two types of action tremors, i.e., emission of the continuous /s/ and the whistle. Tremors were classified as essential when observed during the execution of both types of tasks and dystonic when not observed during the emission of /s/ and the whistle 5,6.

### Therapeutic interventions

The patients received 2 treatments: percutaneous injection of botulinum toxin type A into the left TA muscle under electromyographic guidance, and oral administration of a beta-adrenergic blocker (propranolol). The order in which the two treatments were performed was determined randomly; the date for each type of treatment was selected, and patients who randomly arrived on that date received the treatment. Botulinum toxin (Dysport - APSEN) was administered at a dose of 15 units, reconstituted to a concentration of 1 unit in 0.1 ml of normal saline. The propranolol total daily dose was 80 mg, administered in the morning and afternoon. The same dose was maintained from the beginning until the end of treatment.

Before the treatments began, the patients’ voices were recorded. Three weeks after each treatment was initiated, patients underwent a second nasofibrolaryngoscopy procedure to confirm the efficacy of the treatment and provide a second voice recording for subsequent perceptual assessments and acoustic analyses. Patients who received botulinum toxin in the first round of treatment (n=7) waited 6 months before beginning a second round of treatment with oral propranolol. Patients who received oral propranolol in the first round (n=9) had a 2-month washout period without treatment before beginning the second round of treatment with botulinum toxin. One of the patients died from an unknown cause before completing the treatment (Figure 1).

### Perceptual and acoustic analyses

Vocal tasks performed by each participant included the sustained emission of the vowel /ε/ at a comfortable and auto-regulated intensity and frequency, recorded before and 3 weeks after the start of each treatment. Patients recorded their voices while seated in a silent environment using a unidirectional headset microphone with a flat response curve positioned 1 cm diagonally from the corner of the mouth and coupled to a desktop computer. Voices were recorded directly into audio editing software (Sound Forge version 4.5, Sony) with the audio input set using the volume control function of the software. Each voice sample was edited in the audio editing software, excluding the start of each emission but retaining approximately the subsequent 3 seconds. A total of 45 voice samples were analyzed, 3 from each of the 15 included patients, before and after each treatment; 20% of the repetitions (9 voices) were also analyzed to verify intra-rater reliability. The vocal emissions underwent two types of analysis, perceptual and acoustic, which were based on the overall degree of vocal deviations and the instability of the sustained long vowel /ε/ on a visual analog scale of 100 points, where 0 indicated no change and 100 indicated the maximum change. The evaluations were made independently; the assessments made by the reviewer with the greater reliability (91.26%) were used in this study (Table 1). The acoustic analysis of the voices was performed with VoxMetria version 2.7h (CTS Informática).

The extracted acoustic parameters of the phonations included jitter, shimmer, and variability of the fundamental frequency in semitones.

### Statistical analysis

The Mann-Whitney test was used to compare pretreatment quantitative variables, including the overall degree of voice change, vocal instability, jitter, shimmer, and variability of the fundamental frequency, between the vocal tremor groups, essential and dystonic. The Wilcoxon test was used to compare the responses to the two treatments based on the perceptual evaluation results obtained before and after treatment for each vocal tremor group. The significance level adopted for this study was 0.05 (5%). Statistical analyses were performed using SPSS v.17 (IBM, Armonk, NY), Minitab 16 (Minitab, State College, PA), and Excel (Office 2010, Microsoft, Redmond, WA).

## RESULTS

Table 1 shows the comparisons of the perceptual evaluation parameters (overall degree of vocal change and vocal instability) and also presents a pretreatment comparison of the parameters of the acoustic assessment (jitter, shimmer, and variability of the fundamental frequency) between patients with dystonic and essential tremors. Statistical analyses revealed no significant differences between the two types of tremors for the parameters assessed.

Table 2 presents the perceptual and acoustic assessment results for patients with essential tremors after treatment, compares the results of each treatment with the pretreatment values, and compares the results of the two treatments. There were no statistically significant differences.

Table 3 presents the perceptual and acoustic assessment results for patients with dystonic tremors after treatment, compares the results of each treatment with the pretreatment values, and compares the results of the two treatments. Statistical analysis showed that the overall level of change, vocal instability, and variability of the fundamental frequency were significantly lower after the administration of botulinum toxin than before the treatment. The use of propranolol led to no statistically significant differences. The differences between botulinum toxin and propranolol in the overall level of vocal change, jitter, and shimmer were nonsignificant. The variability of the fundamental frequency and vocal instability values were significantly lower after the injection of botulinum toxin than after the administration of oral propranolol.

## DISCUSSION

This randomized study compared the responses of the most common types of action-induced vocal tremors, essential and dystonic, to the injection of botulinum toxin into the TA and the oral administration of propranolol.

The classification of essential and dystonic vocal tremors, which are physiologically distinct, was made through evaluations with nasofibrolaryngoscopy during unvoiced tasks. The two types of tremor did not differ in the perceptual evaluation of voiced sounds or in acoustic analysis, based on the parameters evaluated in the pretreatment period (Table 1).

For patients with essential tremor, the comparison of the two treatment methods by perceptual and acoustic analyses revealed no statistically significant differences (Table 2). Similarly, no statistically significant differences were observed before and after treatment. The results for the vocal instability and variability of the fundamental frequency values suggested a better response to propranolol than to botulinum toxin (Table 2). A study using a larger sample of patients could potentially confirm this trend. Warrick et al. 9 reported that vocal parameters in a patient with essential vocal tremor improved after 16 weeks of bilateral administration of 2.5 units of botulinum toxin into the TA muscles. In the same year, however, the authors published a prospective study comparing unilateral and bilateral botulinum toxin injections in patients with essential tremor. In that study, only 3 of the 10 patients who received bilateral injections and 2 of 9 patients who received unilateral injections experienced improvements in the acoustic measurements of tremor. The authors also reported that 8 of the 10 patients requested a reinjection of the botulinum toxin because of vocal effort; however, the authors emphasized the need for further studies to clarify the subgroups likely to benefit from this treatment. Adler et al. 12 evaluated 13 patients with essential vocal tremor who received 1.25 units, 2.5 units, or 3.75 units of botulinum toxin in each vocal cord. The authors found a progressive improvement with increasing dose, based on perceptual and acoustic parameters and videolaryngostroboscopy. Kendall and Leonard 15 presented a series of 4 patients with vocal tremors who received botulinum toxin injections in the TA and interarytenoidal muscles; the results of injections into only the TA muscle were not beneficial, as observed by laryngeal dystonia. The authors concluded that the combination of injections into the two muscles could improve the voice quality of low-risk patients. This method could provide an alternative treatment for patients but may have the potential for aspiration. Gurey et al. 16, in a retrospective analysis of 16 patients with essential tremors who had received botulinum toxin in both vocal muscles, observed a symptomatic improvement and reduction in tremor amplitude in the larynx in all patients. Of these patients, 50% had not responded well to therapy with oral medications. The authors concluded that botulinum toxin was useful in patients with essential tremors and that the doses and application sites should be individualized according to tremor characteristics, symptoms, and adverse effects. Lorenz and Deuschl 17 discussed the traditional view that essential tremor has no well-defined cause and mechanism because it is not an isolated condition. They stated that the heterogeneity of the disease is caused by gaps in diagnostic criteria and the diversity of neurological manifestations that may be associated with tremors. Controlled studies with larger numbers of patients may help define the best treatment. However, the possibility of satisfactory responses to either treatment justifies the use of both in clinical practice. Patients selected for the current study spontaneously sought out medical help with a complaint of vocal tremor, which is not common in cases of essential tremors in general. Although vocal tremor is not uncommon in these patients, it is usually less important to the patient. In addition, the majority of patients excluded during the survey were from the essential tremor group. Thus, the patients in the essential tremor group shown in Table 2 showed better absolute values in terms of the overall levels of change, vocal instability and variability of F_0_. Maybe due to the small number of patients, we did not have statistically significant results in the essential vocal tremor group, which is a limitation of this research. However, compared with the absolute values in Table 3, where there was a statistically significant difference, lower values were observed in the essential tremor group (Table 2). Studies with a higher number of patients are desirable.

In patients with dystonic tremors, the assessment of the overall level of vocal changes showed a tendency towards a statistically significant difference, with minor changes after the use of botulinum toxin compared to propranolol (Table 3). A study with a larger number of patients might result in a significant difference. Vocal instability was significantly lower after the administration of botulinum toxin than after the administration of propranolol (Table 3). The difference in the variability of the fundamental frequency between the two treatments in patients with dystonic tremors was statistically significant; botulinum toxin injection produced better results (Table 3). This parameter is highly associated with tremor because it measures variations in the fundamental frequency, and its decrease is related to improvements in tremor. Comparisons of assessments of perceptual parameters before and after the treatments showed a statistically significant difference for the parameter of vocal instability, with a better response to botulinum toxin injection in patients with dystonic tremors. The comparison of acoustic data before and after the treatments also showed a statistically significant difference in the variability of the fundamental frequency with the use of botulinum toxin injections in patients with dystonic tremors (Table 3). The average variability of the fundamental frequency was very close to normal values. The variability of the fundamental frequency is an objective acoustic parameter that demonstrated differences between treatments and indicated a good response to botulinum toxin injection in patients with dystonic tremors; it must be taken into account in the evaluation and monitoring of patients with vocal tremors. Maronian et al. 3 reported improvements and patient satisfaction in a study of 44 patients with dystonic tremors who received botulinum toxin injections in the lateral cricoarytenoid (21 patients, 48%) or TA muscles (23 patients, 52%); the two groups exhibited similar treatment responses. Injection into the lateral cricoarytenoid muscle could be an option for patients with dystonic tremors, but it may have adverse effects, such as dysphagia, vocal breathiness, and hoarseness. Fasano et al. 18, in a systematic review of 43 publications and 487 patients, reported difficulty in evaluating the effectiveness of treatments for different types of vocal tremors. The interventions were numerous, with oral medications, injectable medications, and stimulants used for different types of tremors, with highly variable results that depended on dosage, application site, and tremor distribution. Most studies investigated the application of botulinum toxin, generally finding improvements in tremors mainly of the head and vocal cords. The authors concluded that randomized controlled trials were needed.

The present study, although randomized, was not double-blinded for ethical reasons because of the impossibility of placebo injections into the muscle. However, the subjective perceptual evaluations and objective acoustic evaluations were blinded. The evaluator did not know the patients and was also unaware of the time period and treatment corresponding to each voice recording. The comparison of the two types of tremors showed that they do not differ in perceptual or acoustic evaluations but did respond differently to treatment with botulinum toxin and propranolol. The response of dystonic tremors to botulinum toxin injections was statistically significant in perceptual and acoustic assessments; this was not observed with essential tremors. Hence, it is important to establish the correct vocal tremor diagnosis in order to offer the proper treatment to the patient and to determine an informed prognosis. The specific measures of vocal instability used here were more sensitive than other parameters for detecting differences, even in a small group of patients; thus, we recommend that these measures be prioritized among the general perceptual and acoustic measures.

Dystonic and essential vocal tremors are unusual, although they are the most common types of vocal tremors. The fact that they affect elderly patients complicates the use of beta-adrenergic blocking drugs. In this study, 7 patients were excluded because of the impossibility of using this treatment (Figure 1). Further studies with a larger number of patients comparing different doses of botulinum toxin, bilateral and unilateral administration, injections in other intrinsic muscles, and the administration of other oral drugs may supply new data to support clinical applications in patients with vocal tremors.

## CONCLUSIONS

1. Dystonic and essential tremors differ in their responses to treatments.
2. Dystonic tremor responds significantly to the injection of botulinum toxin but not to the use of oral propranolol.
3. Essential tremor does not respond significantly to either treatment.

## AUTHOR CONTRIBUTIONS

Guglielmino G was responsible for the analysis of videos, interview of patients and preparation of the manuscript. De Biase NG was responsible for the study guidance and conception, organization and review of the manuscript. Villanova LC was responsible for the manuscript review. Moraes BT was responsible for the analysis of the videos and manuscript review. Padovani M was responsible for the analysis of the voices.

## Figures and Tables

**Figure:**
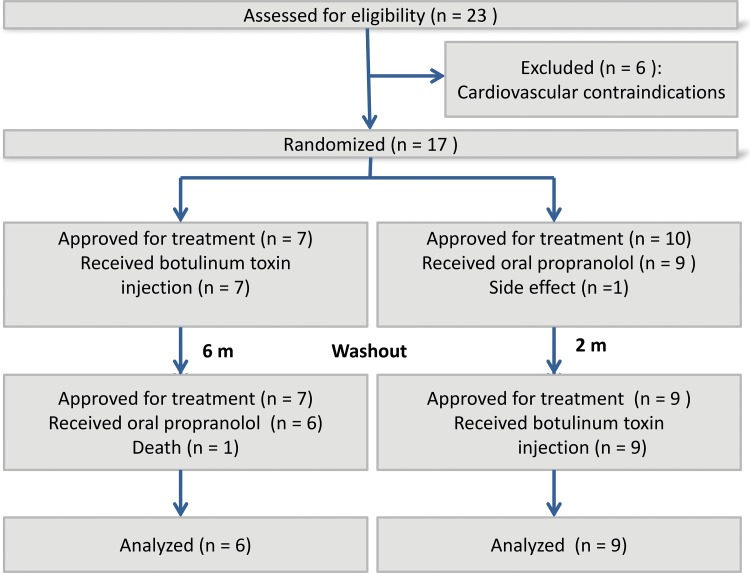
Flow diagram of the phases in the parallel randomized trial of the two groups of vocal tremor patients.

**Table 1:** Pretreatment comparison of perceptual and acoustic parameters between patients with dystonic and essential tremors.

**Overall level of change**	**Dystonic tremor**	**Essential tremor**
N	10	5
Mean	78.0	92.0
SD	15.5	8.4
*P* value	0.097	
**Instability**	**Dystonic tremor**	**Essential tremor**
Mean	79.5	90.0
SD	13.8	12.2
*P* value	0.130	
**Jitter**	**Dystonic tremor**	**Essential tremor**
Mean	3.17	4.76
SD	4.65	4.69
*P* value	0.806	
**Shimmer**	**Dystonic tremor**	**Essential tremor**
Mean	21.26	24.56
SD	14.08	14.69
*P* value	0.624	
**Variability of F_0_**	**Dystonic tremor**	**Essential tremor**
Mean	11.00	11.60
SD	4.78	4.98
*P* value	0.901	

N, number of participants; SD, standard deviation; F0, fundamental frequency.

**Table 2:** Perceptual and acoustic assessment results for patients with essential tremors before and after the administration of botulinum toxin and propranolol, comparing the results of each treatment method with pretreatment values and comparing the results of the two treatments.

		N	Mean	Standard deviation	*P1* value	*P2* value
Overall Level of change	Pre	5	92.0	8.4	- x -	
	BT	5	82.6	18.0	0.336	0.345
	PP	5	71.6	33.1	0.109	
Vocal instability	Pre	5	90.0	12.2	- x -	
	BT	5	73.0	22.8	0.104	1.000
	PP	5	70.8	29.9	0.068	
Jitter	Pre	5	4.76	4.69	- x -	
	BT	5	3.90	3.82	0.686	0.893
	PP	5	4.10	5.72	0.500	
Shimmer	Pre	5	24.56	14.69	- x -	
	BT	5	20.41	10.24	0.500	0.893
	PP	5	21.21	19.18	0.686	
Variability of F_0_	Pre	5	11.60	4.98	- x -	
	BT	5	8.20	3.42	0.225	0.893
	PP	5	9.00	7.07	0.077	

Pre, pretreatment; BT, botulinum toxin; PP, propranolol; F0, fundamental frequency; N, number of participants; *P1*, comparison of each treatment method before and after treatment; *P2*, comparison between treatments.

**Table 3:** Perceptual and acoustic assessment results for patients with dystonic tremors before and after the administration of botulinum toxin and propranolol, comparing the results of each treatment method with pretreatment values and comparing the results of the two treatments.

		N	Mean	Standard deviation	*P1* value	*P2* value
Overall level of change	Pre	10	78.0	15.5	- x -	
	BT	10	60.5	16.9	0.031*	0.059
	PP	10	77.5	19.3	0.953	
Vocal instability	Pre	10	79.5	13.8	- x -	
	BT	10	50.0	18.3	0.007*	0.024*
	PP	10	78.3	20.5	0.959	
Jitter	Pre	10	3.17	4.65	- x -	
	BT	10	2.94	4.76	0.386	0.508
	PP	10	4.17	5.45	0.959	
Shimmer	Pre	10	21.26	14.08	- x -	
	BT	10	17.45	13.13	0.386	0.386
	PP	10	22.64	17.70	0.799	
Variability of F_0_	Pre	10	11.00	4.78	- x -	
	BT	10	5.40	3.34	0.011*	0.050*
	PP	10	10.70	6.09	0.683	

Pre, pretreatment; BT, botulinum toxin; PP, propranolol; F0, fundamental frequency; N, number of participants; *P1*, comparison of each treatment method before and after treatment; *P2*, comparison between treatments; * *p*<0.050.
